# Development and characterization of ferret *ex vivo* tracheal injury and cell engraftment model

**DOI:** 10.3389/fmed.2023.1144754

**Published:** 2023-04-11

**Authors:** Vitaly Ievlev, Albert C. Pai, Drew S. Dillon, Spencer Kuhl, Thomas J. Lynch, Kyle W. Freischlag, Caitlyn B. Gries, John F. Engelhardt, Kalpaj R. Parekh

**Affiliations:** ^1^Department of Anatomy and Cell Biology, Carver College of Medicine, University of Iowa, Iowa City, IA, United States; ^2^Department of Cardiothoracic Surgery, Carver College of Medicine, University of Iowa Hospitals and Clinics, Iowa City, IA, United States; ^3^Protostudios, Carver College of Medicine, University of Iowa, Iowa City, IA, United States

**Keywords:** explant, engraftment, tissue-transwell, trachea, ferret, injury model, regeneration, airway

## Abstract

The field of airway biology research relies primarily on *in vitro* and *in vivo* models of disease and injury. The use of *ex vivo* models to study airway injury and cell-based therapies remains largely unexplored although such models have the potential to overcome certain limitations of working with live animals and may more closely replicate *in vivo* processes than *in vitro* models can. Here, we characterized a ferret *ex vivo* tracheal injury and cell engraftment model. We describe a protocol for whole-mount staining of cleared tracheal explants, and showed that it provides a more comprehensive structural overview of the surface airway epithelium (SAE) and submucosal glands (SMGs) than 2D sections, revealing previously underappreciated structural anatomy of tracheal innervation and vascularization. Using an *ex vivo* model of tracheal injury, we evaluated the injury responses in the SAE and SMGs that turned out to be consistent with published *in vivo* work. We used this model to assess factors that influence engraftment of transgenic cells, providing a system for optimizing cell-based therapies. Finally, we developed a novel 3D-printed reusable culture chamber that enables live imaging of tracheal explants and differentiation of engrafted cells at an air-liquid interface. These approaches promise to be useful for modeling pulmonary diseases and testing therapies.

**Graphical abstract:**
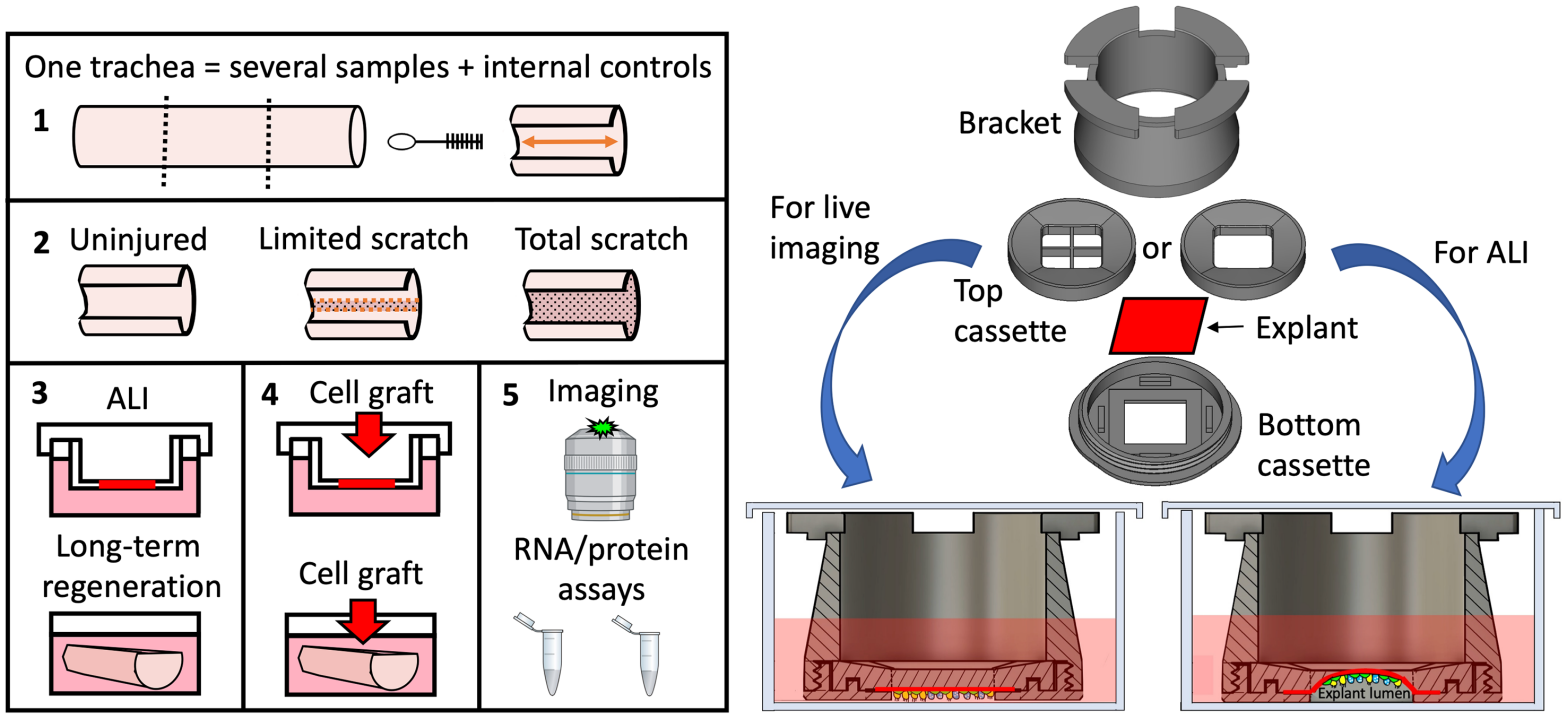
**1**,**2**. We describe here a method for differential mechanical injury of ferret tracheal explants that can be used to evaluate airway injury responses ex vivo. **3**. Injured explants can be cultured at ALI (using the novel tissue-transwell device on the right) and submerged long-term to evaluate tissue-autonomous regeneration responses. **4**. Tracheal explants can also be used for low throughput screens of compounds to improve cell engraftment efficiency or can be seeded with particular cells to model a disease phenotype. **5**. Lastly, we demonstrate that ex vivo-cultured tracheal explants can be evaluated by various molecular assays and by immunofluorescent imaging that can be performed live using our custom-designed tissue-transwell.

**1**,**2**. We describe here a method for differential mechanical injury of ferret tracheal explants that can be used to evaluate airway injury responses ex vivo. **3**. Injured explants can be cultured at ALI (using the novel tissue-transwell device on the right) and submerged long-term to evaluate tissue-autonomous regeneration responses. **4**. Tracheal explants can also be used for low throughput screens of compounds to improve cell engraftment efficiency or can be seeded with particular cells to model a disease phenotype. **5**. Lastly, we demonstrate that ex vivo-cultured tracheal explants can be evaluated by various molecular assays and by immunofluorescent imaging that can be performed live using our custom-designed tissue-transwell.

## Introduction

In recent years the field of airway biology has seen a surge in new imaging and culture methods. Novel techniques such as precision-cut lung slices (1) and lung-on-a-chip models (2, 3) are gaining popularity among researchers because they account for 3-D complexity and cell-type heterogeneity of the airways. In addition, *in vitro* monocultures of airway epithelial progenitors enable researchers to precisely manipulate and image cells. However, these methods cannot account for the complex cellular crosstalk that drives non-cell autonomous effects originating from cells in the airway mesenchyme, cartilage, submucosal glands (SMGs), and elsewhere. Airway tissue complexity is reflected by the plethora of cell types discovered by recent single-cell RNA sequencing studies (4–7). Although *in vivo* experimentation remains the gold standard in terms of biological relevance of the findings, many of the precise manipulations and imaging methods are costly and either challenging or impossible to carry out in living animals. *Ex vivo* tissue culture approaches overcome some of the limitations of *in vivo* and *in vitro* models and also provide an independent means of validating the experimental results.

In the current study, we characterized a ferret tracheal explant model of injury and cell engraftment. As opposed to mice, rats, and rabbits, for which tracheal explants were described previously (8–10), ferrets are useful, because they have extensively developed submucosal glands (SMG) throughout cartilaginous airways, which is also the case in humans. This is critical because these structures contribute significantly to airway regeneration (11, 12). Even though some other species (i.e., pigs and sheep) also have well developed SMGs, ferrets are exceptionally useful as a model organism due to relatively low costs for raising and housing them. Additionally, existing transgenic ferret lines can be used for lineage tracing (e.g., a fluorescent reporter ROSA26-Tomato/EGFP and several Cre^ERT2^ driver lines are available) (13).

In this work we developed protocols for *ex vivo* culture, injury, cell engraftment, and imaging of ferret tracheal explants. In addition, we designed and tested a tissue culture chamber for explants that enables separation of the luminal and adventitial sides of the explant to create an air-liquid interface (ALI). We optimized *ex vivo* culture conditions to stimulate regeneration of the surface airway epithelia by both the surface basal cells and the glandular progenitors such as myoepithelial cells (MECs). Our *ex vivo* model of ferret trachea allows us to screen for optimal engraftment conditions at a much lower cost than that associated with *in vivo* methods. As a proof of principle, we show that both partial and complete removal of the tracheal explant epithelium stimulates engraftment of Tomato+ primary ferret airway basal cells. These findings are important because patients with tracheal stenosis or genetic airway diseases such as cystic fibrosis (*CF*) could potentially be treated by engraftment of gene-corrected, autologous cells either directly into the patient or into a tracheal scaffold. We additionally show that this *ex vivo* model can be used to study transcriptional changes in the tracheal epithelium, both after injury and after transgenic cell engraftment, and that it can be used to generate air-liquid interphase cultures that are compatible with live imaging.

## Materials and methods

### *Ex vivo* tracheal brush injury

We used adult wild-type (WT) ferret tracheas for scratch injury assays *ex vivo*. Shortly after the animal was euthanized, the trachea was dissected and cut open longitudinally on the membranous side while the cartilaginous side was brushed with a stiff nylon brush (Justman Brush Company, cat#415140, d = 2 mm). Specifically, injury was performed either by making 3 overlapping streaks along the proximal-distal axis (limited injury) or by extensively brushing in all directions (total injury). The explants were then cultured in F-medium [3:1 (v/v) DMEM (Invitrogen): F-12 Nutrient Mixture (Gibco), 7.5% FBS, 1% Penicillin/Streptomycin, 125 ng/ml epidermal growth factor (Invitrogen), 25 μg/mL hydrocortisone (Sigma-Aldrich), 5 μg/mL insulin (Sigma-Aldrich), 0.01 mg/mL Gentamicin, 0.1% amphotericin B, 11.7 μM cholera toxin (Sigma-Aldrich) and 10 μmol/L Y-27632 (Tocris)] and pulsed with a nucleotide analog – 10 μM EdU (5-ethynyl-2′-deoxyuridine, Thermo Fisher Scientific C10340) on specified days after injury.

### Whole-mount ferret trachea staining

At various experimental timepoints the explants were fixed in 4% paraformaldehyde or 10% neutral buffered formalin for 2 h at room temperature on a rocker, then washed three times in PBS for 20 min each. For staining of proteins that require antigen retrieval, samples were additionally incubated in citrate buffer (10 mM sodium citrate, 0.05% Tween 20, pH = 6.0) at 55°C overnight. The samples were then washed in PBS three times and incubated in blocking buffer (20% donkey serum, 0.3% Triton X-100, and 1 mM CaCl_2_ dissolved in PBS, pH 7.6), either overnight at 37°C or for 24–72 h at 4°C. Tissues were rinsed in PBS, after which EdU was detected using the Click-iT™ EdU Cell Proliferation Kit for Imaging (Thermo Fisher Scientific), in accordance with the supplied protocol. The tissues were then washed three times for 20 min each in PBS and incubated with primary antibodies (dissolved in diluent buffer: 1% donkey serum, 0.3% Triton X-100, and 1 mM CaCl_2_ in PBS, pH 7.6) for 18–24 h at 37°C with agitation (see Table 1). The tracheas were then washed three times for 20 min in PBS and incubated with secondary antibodies (dissolved in diluent buffer) for 24 h at 37°C, with agitation. Next, the tissues were washed three times for 20 min in PBS and submerged in Ce3D tissue clearing solution (Biolegend cat# 427704) for 2 h at RT or overnight at 4°C. Samples were mounted under 0.33 mm coverslips or under 1 mm glass slides and the edges were sealed with Gorilla Glue™ and clamped with binder clips for about 20 min to ensure glue fixation. Tile scan and Z-projection modules of a Zeiss LSM 880 or 980 line-scanning confocal microscope (Carl Zeiss, Germany) were used for imaging of the entire explant.

**Table 1:** Primary antibodies.

Antibody target	Company	Catalog #	Concentration
KRT5	BioLegend	905,901	1:500
KRT15	BioLegend	833,901	1:200
AC-TUB	Cell Signaling Technology	5,335	1:500
CCSP	Millipore Sigma	ABS1673	1:4000
KRT14	Labvision	RB-9020-P1	1:500
KRT14	Thermo Fisher Scientific	MA5-11599	1:300
α-SMA	Abcam	ab7817	1:500
SOX9	Millipore Sigma	AB5535	1:500
KRT8	Origene	BP5007	1:500

### Isolation, culture, and engraftment of primary airway basal cells

#### Isolation of primary cells

Primary surface epithelial cells were isolated from ferret trachea and/or extra-lobar bronchi by a previously described enzymatic digestion method (14). Tissues were digested for 1 h at 37°C with agitation, in 5 mg/ml pronase (Roche) dissolved in F-12 medium (Gibco), to dissociate surface airway epithelial (SAE) cells. Epithelium from the luminal surface of the airways was further detached with a cell scraper. Detached sheets of SAE cells were then passed through a 100 μm strainer to separate cell aggregates and to filter out the debris. Next, we washed the cells twice in an excess of complete DMEM (DMEM with 10% FBS, 1% Penicillin/Streptomycin) to remove all traces of pronase. The SAE cells were then plated on 804G-coated plastic plates. The remaining tissue, which contained SMG cells was minced and incubated for 45 min at 37°C on a rocker in 1X Collagenase/Hyaluronidase (STEMCELL Technologies, Inc.; diluted from 2x in F-12 medium). For every 3 ml of 1x Collagenase/ Hyaluronidase solution, 1 ml of Trypsin/EDTA (0.025% Trypsin, 0.01% EDTA: Thermo Fisher Scientific) was added and samples were incubated for an additional 10 min at 37°C. After the digestion, SMG cells were liberated by pipetting using a 10 ml serological pipette tip. The detached SMG cells were then passed through a 100 μm cell strainer and washed twice in an excess of complete DMEM, and finally plated on 804G-coated tissue culture dishes.

#### Primary cell culture

Primary cells were cultured under the conditions used to propagate airway basal cells (BCs) and SMG progenitors as described previously (14). Ferret primary airway basal stem cells were cultured in PneumacultEX+ with 1% Penicillin/Streptomycin. 2-D cell cultures were grown on tissue-culture plastic pre-coated with filter-sterilized, extracellular matrix (ECM)-enriched 804G-conditioned medium for ≥1 h as described previously (14). Accutase (STEMCELL Technologies, Inc.) was used to detach the cells from the culture plates. During the washes, the cells were centrifuged for 5 min at 500 g force.

### Quantitative PCR analysis

RNA was isolated from brushed off cells using Trizol regent (Ambion) following the manufacturer’s protocol. A High-Capacity cDNA Synthesis Kit (Applied Biosystems) was used to synthesize cDNA following the protocol provided by the manufacturer. qPCR reactions were set up using 10 nM primers, the POWER SYBR master mix (Applied Biosystems), cDNA, and H_2_O. Reactions were run on a CFX Connect Real-Time PCR Detection System (Bio-Rad). The Delta–Delta-CT method was used to normalize the expression data. Primers are listed in Table 2.

**Table 2:** Ferret qPCR primers.

ITGB1 qPCR primers	Fwd: TGTATACAAGCAGGGCCAAAT Rev.: TCTCTGCTGTTCCTTTGCTAC
ITGA6 qPCR primers	Fwd: CGACCCTTCATCAGAAAGCA Rev.: CTCCATCCATGTCATCCTCAATC
ITGA3 qPCR primers	Fwd: CCGATTCCTGGTGGTGAAG Rev.: GCTCACAGTCGTCCTTGTC
CD44 qPCR primers	Fwd: GTGGAGAAGAATGGTCGTTACA Rev.: GGTGTTGGATGTGAGGATGT
Tomato qPCR primers	Fwd GTATCGGACAGCGCAAAGAACG Rev. CTGGTAGGTACAGCAGCTCATC
LAMA3 qPCR primers	Fwd: CTCAGGCACACAGTACAACA Rev.: GAGTAGGTGCTTCCAAAGTCTAC
MMP9 qPCR primers	Fwd: GAAGCCGACATCGTCATTCA Rev.: CAGGGACCACAACTCTTCATC
SOX9 qPCR primers	Fwd: GTCAACGAGTTCGACCAGTA Rev.: CAGCTGCTCCGTCTTGAT
ACTN-B qPCR primers	Fwd: TGGGACGACATGGAGAAGAT Rev.: CCTGGATGGCCACATACAT

### Image analysis

Merged tile scans were quantified using ImageJ. Epithelial coverage of the explants was scored in ImageJ by dividing the area stained with epithelial markers (KRT5, KRT14, KRT15 or Tomato) over the total area of the explant. The % fraction of actively proliferating SMGs was calculated by dividing the selected areas with discretely high EdU+ cell density by the total area of all the SMGs as defined morphologically by staining with Krt14/5 and/or αSMA and Sox9.

### Statistical analysis

Unless stated otherwise in a figure legend, results are reported as mean +/− SEM, individual dots on the graphs represent biological replicates, and statistical analysis was conducted using Prism version 9 (GraphPad Software) where N equaled the number of independent animals. The statistical tests used are stated in each figure legend. Data were considered significant at *p* < 0.05.

## Results

### Whole-mount staining reveals fine details of anatomy of the ferret trachea

**Figure 1:**
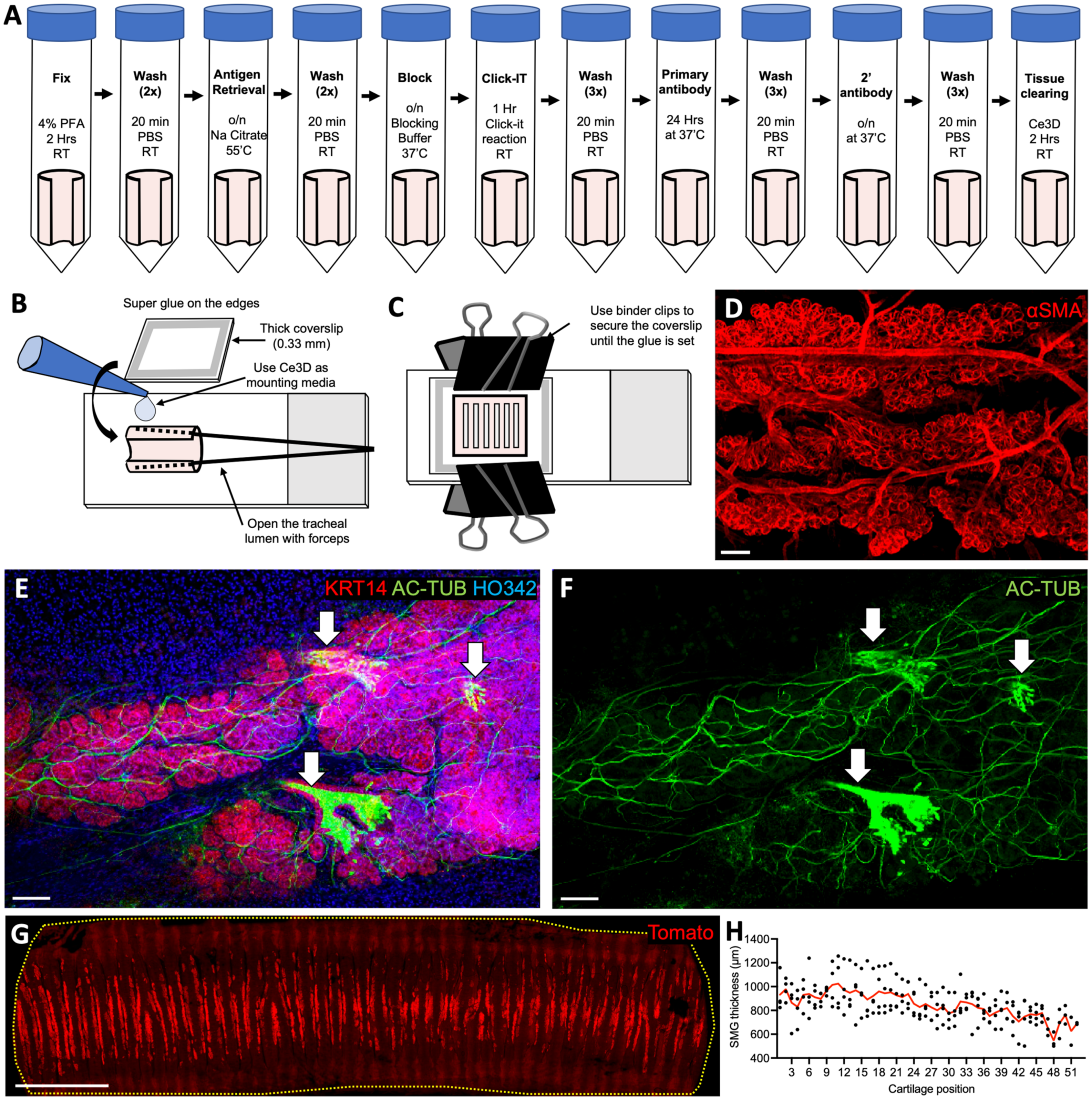
Whole-mount staining of ferret tracheal explants reveals SMG structure and distribution. **(A)** Protocol for whole-mount staining of ferret tracheal explants (open pink cylinders on the schematic). **(B,C)** Steps in mounting ferret tracheal explants. **(D–F)** Confocal maximum intensity projections of the SMGs from ferret tracheas stained on whole-mount. **(D)** αSMA staining, showing vascularization and SMG structure. **(E,F)** AC-TUB staining, showing glandular innervation and ciliated ducts (arrows). **(G)** Confocal maximum intensity projection of an adult ROSA-TG ferret trachea where Tomato expression is enriched in SMGs. **(H)** Quantification of the SMG thickness in relation to cartilage position. Red line represents an average value for *N* = 4 ferrets. All scale bars are 100 μm, except in **(G)** the scale bar is 1 cm.

Whole-mount tissue staining provides opportunities to capture aspects of the cellular architecture and organization that are often lost in 2D tissue sections. Furthermore, because a greater surface area is covered in such preparations than in tissue sections, this approach makes it possible to capture cellular events at the whole organ level. This approach is also well suited for capturing rare cell types and structures in the organ of choice, for example, the ionocytes in the airway epithelium. We developed an accessible and cost-effective method for whole-mount staining, clearing, and embedding of the ferret trachea (Figures 1A–C) that enables clear visualization of SMGs (Figures 1D–F). Specifically, we found that between each cartilage ring, ferrets have two rows of SMG acini, each perfused with blood vessels (20–25 μm in diameter) that branch laterally to the acini bundles (Figure 1D). Additionally, SMGs appear to be the most innervated structure in the trachea, with a dense network of thick Ac-Tubulin+ nerve bundles surrounding the acini (Figures 1E,F). This whole-mount staining approach can be used to evaluate anatomical structures over a large tissue area. For example, we imaged tracheal fragments 7–7.5 cm in length, from four animals, covering 100% of the width and about 90% of the length of an adult ferret trachea, to evaluate the SMG distribution. Our examination of ROSA-TG transgenic ferrets revealed that SMGs are located only on the ventral side of the trachea, between the cartilage rings, and that their abundance declines slightly along the distal axis (Figures 1G,H). In summary, our whole-mount staining and confocal microscopy experiments reveal the complexities of the structure, innervation, and vascularization of the trachea.

### F-medium stimulates induction of KRT14 at the wound edges and proliferation of the SAE and SMGs during regeneration after tracheal injury *ex vivo*

Next, we determined the culture conditions conducive to robust SAE repair and to proliferation of SMG progenitors after injury. We initially tested two media: DMEM containing antibiotics and antifungal agents; and F-medium, which contains DMEM, F12, 7.5% FBS, and other additives (see the methods for details). DMEM was chosen for its simplicity because we hypothesized that tracheal explants can produce everything that they require for prolonged survival and repair after injury. F-medium was chosen because it is permissive for the survival of both epithelial and mesenchymal cells when used in colony formation efficiency (CFE) assays of airway basal cells that are grown on 3 T3-J2 fibroblasts (15, 16).

**Figure 2:**
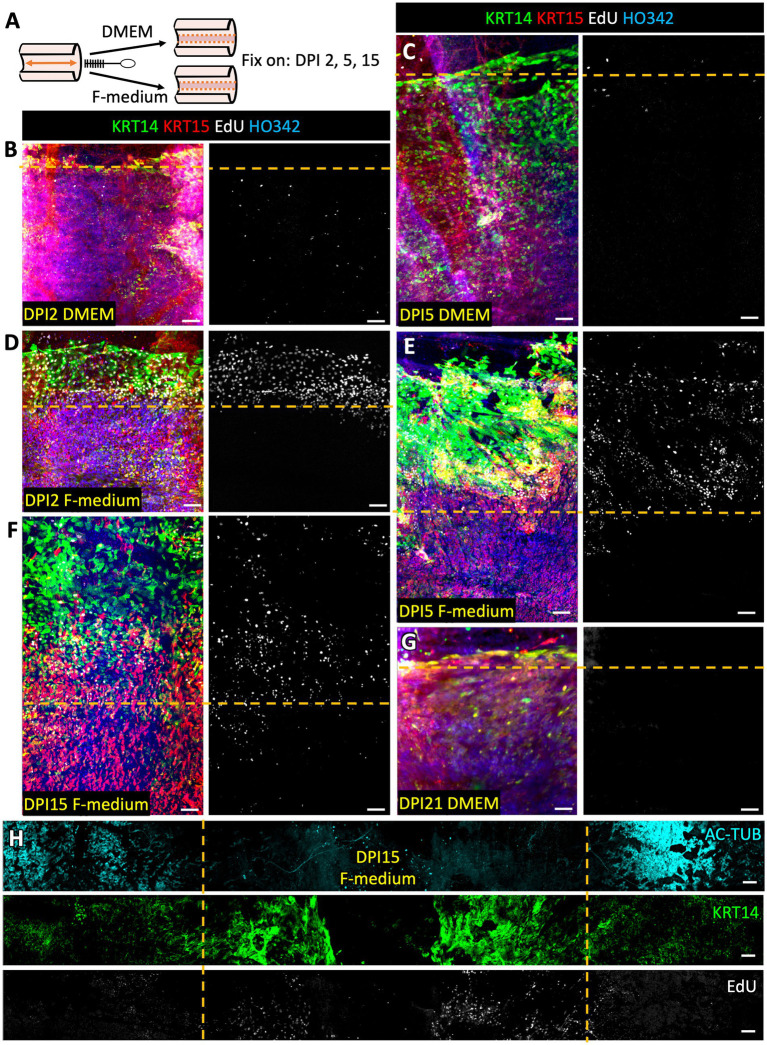
F-medium with Rock-inhibitor stimulates SAE regeneration, upregulation of KRT14 and proliferation at the wound edges in injured tracheal explants. **(A)** Experimental design: The SAE of WT ferret tracheal explants was injured by limited brushing and cultured in DMEM or in F-medium. Samples were fixed and stained on the specified days post injury (DPI). DPI2 samples were cultured with EdU for during the entirety of the experiment, whereas all other samples were pulsed with EdU for 24 h on DPI3. **(B,C,G)** Representative confocal micrographs of the explants cultured in DMEM. **(D–F,H)** Representative confocal micrographs of the explants cultured in F-medium. Results were reproduced using *N* ≥ 3 ferrets. Orange dashed lines indicate the approximate boundaries of the initial scratch. Scale bars are 100 μm.

The tracheal lumen of ferrets was subjected to mechanical injury using a stiff 2-mm-wide nylon brush. This left a gap in the SAE that was partially repaired by basal progenitor cells over the course of 15 days (Figure 2A). We observed that in basal cells KRT14 was upregulated at the leading edges of the scratch (orange dashed lines in Figures 2B–H). This is consistent with the observations that KRT14 is upregulated in different animal models of airway injury, for example with SO_2_, Cl_2_, naphthalene, and polidocanol as well as in human airway diseases, such as idiopathic pulmonary fibrosis (IPF), restrictive allograft syndrome (RAS), and bronchiolitis obliterans syndrome (BOS) (17–21). KRT14 is not the only airway injury response marker, but it has been one of the more robust and consistent injury-induced gene markers that our laboratory has focused on in the past (16–18). Cellular changes such as upregulation of KRT14 and cell proliferation, as well as altered cell morphology at the wound edges, were more pronounced in F-medium than in DMEM on days 2, 5 and 15 (Figures 2B–G). These findings suggest a more robust injury response in F-medium. Additionally, in the explants cultured in F-medium but not in those cultured in DMEM, a wide band of cells along the scratch boundary incorporated EdU, indicating that they were actively proliferating. Explants cultured in F-medium covered most of the denuded scratch by day 15, whereas those cultured in DMEM showed no signs of scratch re-epithelization by day 21 (Figures 2F,G). After 15 days of recovery while submerged in F-medium, the regenerated epithelium had not differentiated into ciliated cells; however, ciliated cells were retained at the uninjured periphery of the scratch (Figure 2H). We conclude that in the ferret *ex vivo* tracheal injury model F-medium is better suited than DMEM for regeneration studies.

**Figure 3:**
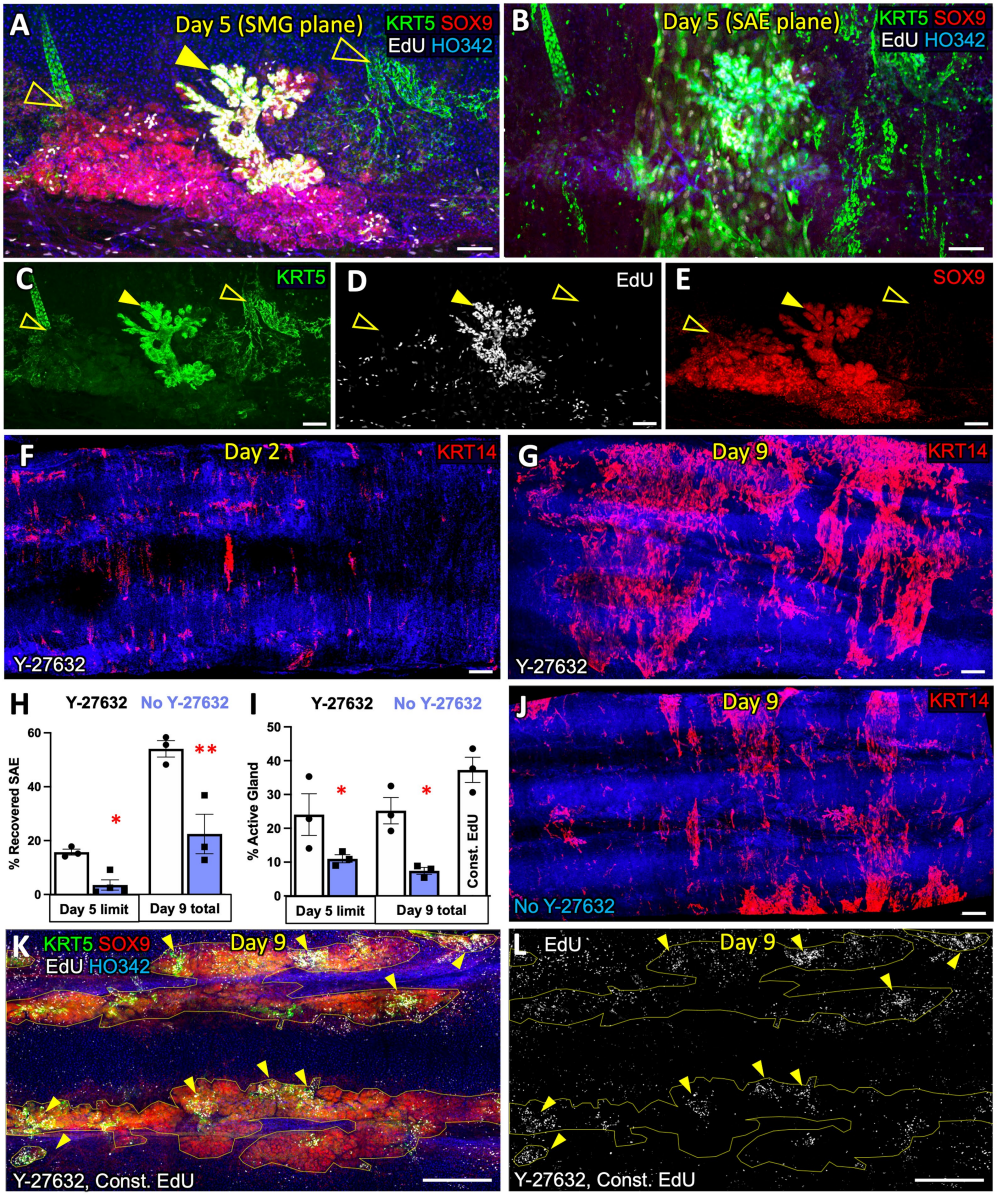
F-medium with ROCK-inhibitor stimulates SAE regeneration by glandular progenitors in ferret trachea *ex vivo*. **(A)** Confocal micrograph of a ferret tracheal explant cultured in F-medium with the ROCK-inhibitor Y-27632, after total brush injury and a 24-h EdU pulse on DPI3. The image shows the diversity of SMG phenotypes providing examples of an actively proliferating gland (solid arrow) and two glands with fewer proliferating cells (unfilled arrows). **(B)** Confocal micrograph focused on the z-plane at the SAE, superficial to the SMGs in panel **(A)**. **(C–E)**, Individual channels of the micrograph in panel **A**, showing **(C)** KRT5, **(D)** EdU, and **(E)** SOX9 expression. **(F,G)** Confocal micrographs of explants subjected to total brush injury, imaged on **(F)** DPI2 and **(G)** DPI9. **(H)** Quantification of SAE recovery, shown as a percentage of the surface area covered with KRT14+ SAE. **(I)** Quantification of the % of SMG area assigned to actively proliferating glands (with high EdU+ cell density). **(J)** Representative micrograph of an explant imaged on DPI9 when cultured in F-medium without Y-27632. **(K,L)** Representative max intensity projections of the SMG plane of the explants that were fully brushed and cultured in constant presence of EdU for 9 days. Graphs show mean +/− SEM, *N* ≥ 3 explants from independent ferrets. Significance was determined by 2-way-ANOVA, Holm-Sidak multiple comparison test. *p < 0.05, **p < 0.01. Scale bars in **(A–E)** are 100 μm; all other scale bars are 500 μm.

The Rho Kinase inhibitor Y-27632 is a standard component of F-medium and is frequently used in culturing airway stem cells. We evaluated the effects of Y-27632 on SAE wound closure and SMG proliferation in our *ex vivo* tracheal injury model. When examining the SMGs following complete removal of the SAE by brushing, we noticed three distinct glandular phenotypes: “passive”, “active”, and “compromised” (Figure 3A). When explants were pulsed with EdU for 24 h on day-3 post injury (DPI3) and fixed on DPI5, “passive” glands contained few EdU+ cells whereas “active” glands contained many and this often coincided with increased KRT5 staining (Figures 3C,D). On DPI5 after total injury (extensive luminal brushing), “active” glands were typically found below KRT5+/EdU+ epithelium on the tracheal surface, suggesting that SAE regeneration involves migration of progenitors from these glands to the airway surface (Figure 3B). Glands of the “compromised” category were relatively rare, comprising <5% of the total gland volume; they incorporated no EdU and were negative for SOX9 (SMG marker, Figure 3E). This gland type most likely represents the SMGs that are no longer viable but retain keratin remnants, and thus also the distinct glandular morphology. We observed that the injured explants cultured in F-medium with Y-27632 contained higher percentages of ‘active’ glands (24.6% +/− 3.3% vs. 9.2% +/− 1.1%) and had more extensive SAE re-epithelialization (15.7% +/− 1.1% vs. 3.5% +/− 1.9% on DPI5 and 54.1% +/−3.0% vs. 22.5% +/− 7.3% on DPI9) than those cultured in F-medium without Y-27632 (Figures 3F–I). We conclude that Y-27632 stimulates SAE regeneration by glandular progenitors, and therefore selected F-medium with Y-27632 as the main culture method for subsequent experiments. Interestingly, most of the SMGs did not actively proliferate even when the explants were continuously cultured in the presence of EdU for 9 days after a total surface brushing. Only 37.3% +/−3.7% of the glands were actively proliferating (Figures 3I,K,L). If this phenomenon holds true *in vivo*, this has interesting implications as a natural mechanism to prevent replicative exhaustion of the glandular SC niche.

### Cell engraftment onto a ferret tracheal explant is facilitated by airway epithelial injury

**Figure 4:**
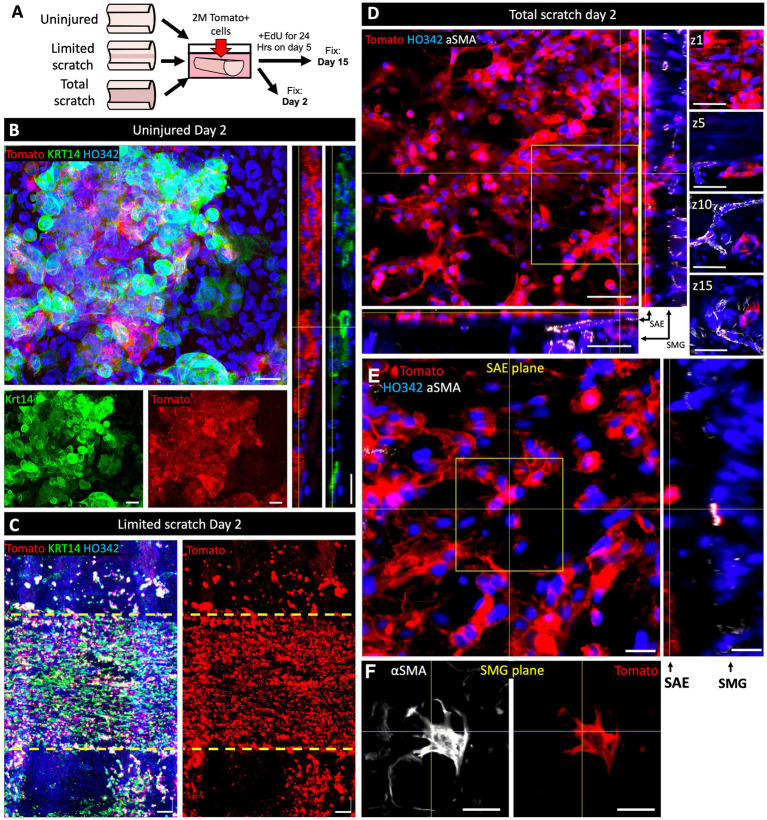
Cell engraftment on ferret tracheal explants is facilitated by epithelial injury. **(A)** Experimental design: Ferret tracheal explants, ~7 mm in length, were either left uninju‑red, partially or completely de-epithelialized by scratching the lumen with a stiff 2-mm nylon brush. Explants were then submerged in F-medium and seeded with 2*10^6^ p6 Tomato+ primary ferret airway basal cells. Samples were fixed on day 2 or day 15 after engraftment of Tomato+ cells. For the day 15 explants, a 24-h pulse of EdU was administered on day 5. **(B–E)** Confocal micrographs of **(B)** uninjured, **(C)** partially brushed, or **(D,E)** fully brushed explants on day 2 after cell engraftment. Insets in panels **(B–E)** show split channels or orthogonal views and different *z*-planes of the boxed regions. Images are representative of *N* = 3 independent experiments. Scale bars in all panels other than **(D)** are 20 μm; in **(D)** they are 50 μm.

**Figure 5:**
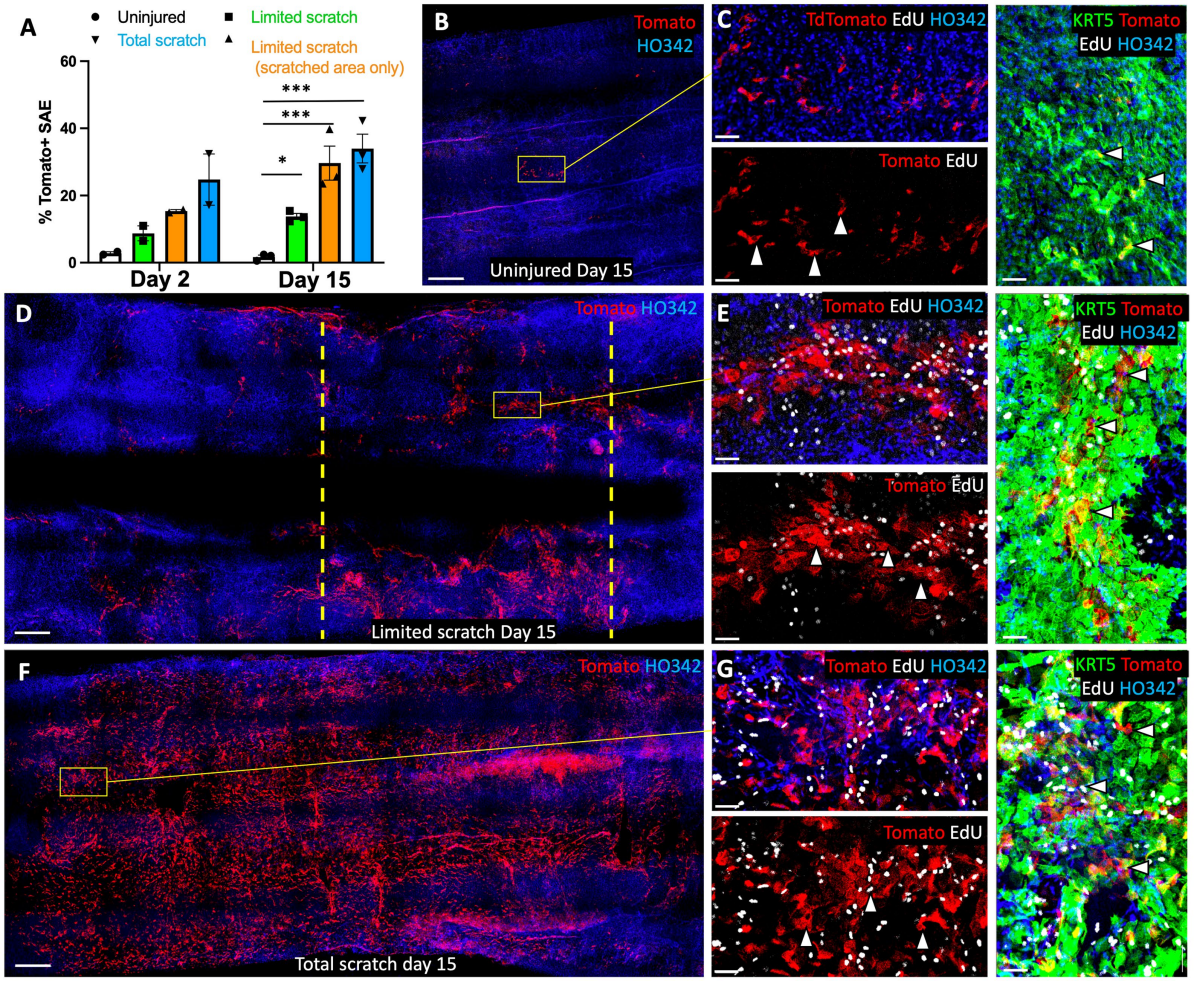
Retention of engrafted cells on ferret tracheal explants is facilitated by epithelial injury on. This experiment was performed as outlined in Figure 4, and all images show the samples on day 15 post engraftment. **(A)** Quantification of explant lumen coverage with Tomato+ SAE on day 2 and day 15 after engraftment. **(B–G)** Confocal micrographs of explants that were **(B,C)** uninjured, or underwent **(D,E)** partial injury or **(F,G)** complete injury, on day 15 after cell engraftment. Composite panels showing KRT5 staining in **(C,E,G)** are rotated 90° clockwise. Dashed lines indicate the boundaries of the scratch. Graph shows Mean +/− SEM, *N* = 3 independent experiments. Significance was determined by one-way ANOVA, Tukey multiple comparison test (*** *p* < 0.001, ns - not significant). Scale bars in **(B,D,E)** are 500 μm; in **(C,E,G)** scale bars are 50 μm.

An important area of interest for cell therapy applications is the efficiency of transgenic cell engraftment (22, 23). With this in mind, we tested the ability of Tomato+ primary basal cells from ferrets to engraft onto intact, partially scratched, and fully scratched WT ferret tracheal explants. We hypothesized that reduced competition with endogenous airway epithelium would facilitate the engraftment of transgenic cells and therefore the efficiency will be higher in the context of total and partial injury versus uninjured explants. We tested this by seeding tracheal explants with 2*10^6^ Tomato+ passage-6 (p6) ferret surface airway basal cells immediately after partial or complete brush injury or onto uninjured control explants (Figure 4A). After the lumen was seeded with Tomato+ cells, the tissues were cultured for 2 or 15 days in F-medium containing Y-27632. We observed that infrequent patches of Tomato+ cells, that engrafted onto uninjured explants on DPI2, stained for KRT14 more intensely than the surrounding Tomato-native SAE (Figure 4B). This outcome was expected because KRT14 is rapidly induced in cultured SAE basal cells (16). In the explants with partially injured SAE, Tomato+ cells were incorporated primarily into the injured regions on day 2 (Figure 4C). In the explants with fully brushed surface after a total injury, SAE Tomato+ cells were incorporated throughout the surface as well as into the SMG ducts on day 2 (Figure 4D); Tomato+ cells were also incorporation into the SMG acini and their differentiation into αSMA+ MECs was observed, but this was very rare (Figure 4E). On day 15, Tomato+ SAE coverage was similar to that on day 2, and it was the highest in the explants subjected to a total injury, where engrafted cells covered 34.0% +/− 4.3% of the explant lumen (Figure 5A). In explants that received a limited scratch injury before cell engraftment, Tomato+ cells were incorporated mostly into the brushed region covering 29.6% +/− 5.1% of the scratch and 13.8% +/− 0.9% of the total explant. Uninjured explants incorporated the fewest Tomato+ cells in the SAE, covering 1.7% +/− 0.6% of the lumen (Figures 5A–C). These findings demonstrate that an injury that creates space for stem cells to attach prior to engraftment can improve their attachment efficiency. Staining of the explants with KRT5 and EdU click chemistry revealed that at least some of the engrafted cells were EdU+/KRT5+ (Figures 5D–G), suggesting that they were able to divide after engraftment which was evident by their incorporation of EdU that was pulsed for 24 h on DPI5.

### Mechanical injury and cell engraftment *ex vivo* results in transcriptional changes to the surface airway basal cells

**Figure 6:**
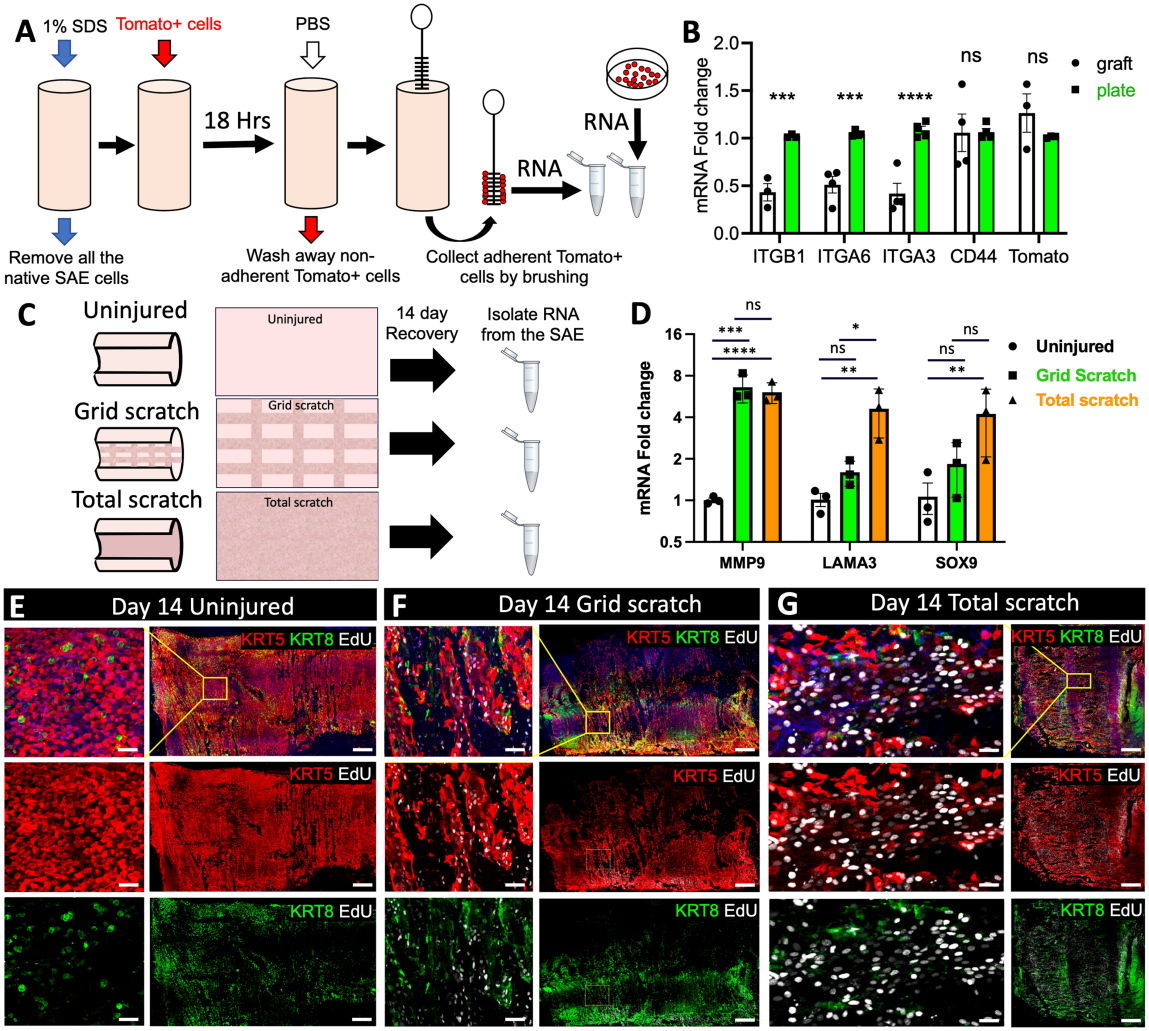
Transcriptional changes in the SAE after mechanical injury and cell engraftment *ex vivo*. **(A)** Experimental design: freshly harvested WT tracheas from adult ferrets were stripped of the native SAE by washing the lumen with 1% SDS. The grafts were then seeded with 10^7^ p6 primary Tomato+ ferret surface airway basal cells using rotating bioreactor as described previously (24). After 18 h, the grafts were washed with PBS 3 times to remove non-adherent cells. The graft lumen was then brushed to collect Tomato+ SAE cells and RNA was isolated from them. Control Tomato+ cells were grown on 804G-coated plates. **(B)** qPCR analysis for the indicated genes in these samples. Graphs show mean +/− SEM, N ≥ 3 independent experiments. **(C)** Experimental design: explants from adult WT ferrets were either left uninjured or differentially injured by total or partial brushing the lumen. The samples were then cultured for 14 days to allow regeneration of the SAE and on day 3 they were pulsed with EdU for 24 h. On day 14 all samples were lightly brushed to remove columnar cells, after which a small slice was cut off and fixed for staining. The rest of the explant was brushed thoroughly to collect basal cells from the lumen which were then processed for RNA isolation and **(D)** qPCR analysis. Graphs show mean +/− SEM. Data are representative of 3 independent experiments. **(E–G)** Representative confocal micrographs of the explant surface epithelium at the time of RNA collection. Significance was determined by 2-way-ANOVA, Tukey’s multiple comparison test in **(D)** and by multiple two-tailed T-tests in **(B)**. ****p < 0.0001, *** *p* < 0.001, ** *p* < 0.01, * *p* < 0.05. Scale bars in **(E–G)** are 500 μm and 50 μm in the enlarged insets.

To better understand the factors that influence the efficiency of cell engraftment, we assessed the differences in the transcription of cell adhesion related genes between Tomato+ cells seeded on a denuded tracheal graft and an ECM-coated cell culture dish. To this end, we removed the SAE from freshly excised tracheas using a series of SDS-containing detergent washes as described previously (24). Then, we engrafted 10^7^ p6 Tomato+ primary surface airway basal cells into a 3-cm ferret tracheal segment using a rotating bioreactor method developed by our group previously for ferret tracheal explant cultures (24). After 18 h, the explants were rigorously washed with PBS to remove any cells that did not fully engraft, and then the luminal surface of the tracheas was brushed to collect Tomato+ cells. RNA from these cells was compared to that of Tomato+ primary basal cells that had been seeded on 804G-coated culture dishes for 18 h (Figure 6A). Subsequent qPCR analysis revealed that expression of ITGB1, ITGA6 and ITGA3 was lower in the cells seeded on tracheal grafts than in those seeded on culture plates (Figure 6B). Expression of CD44, a surface receptor that binds the ECM and promotes cell adhesion was unchanged as was the expression of Tomato, which served as a control to ensure that we had primarily collected the newly engrafted Tomato+ cells and not the native cells of the Tomato-explant (Figure 6B). These data suggest that downregulated integrin expression could account for the decreased efficiency of engraftment of cells on the basal lamina of the tracheal explants versus ECM-coated culture dishes.

Efficient cell engraftment in injured airways is dependent not only on the donor cells but also the injury response of native epithelium that remains present. Therefore, we evaluated how different degrees of explant injury impact the native SAE during regeneration. To this end, we evaluated the cells on the explant surface after 14 days of regeneration following either partial or complete removal of the original SAE by luminal brushings. In the case of partial brush injury, regeneration of the SAE involved both the remaining surface basal cells and SMG progenitors, whereas in the case of total injury, regeneration was attributed primarily to the glandular progenitors. On DPI14 we collected the SAE cells from regenerating explants by brushing the lumen and isolated RNA from them for evaluation by qPCR (Figure 6C). Given that this was a proof-of-principle experiment, we focused on gene targets that we expected to be upregulated in regenerating airway epithelia. Although this method can be used for unbiased screens of transcriptional changes associated with regeneration using single-cell or bulk RNA-sequencing, in this experiment we focused on the expression of three genes. Two of them were MMP9 and LAMA3 that are both associated with ECM deposition and remodeling and are upregulated in epidermal wound healing (25, 26). We also assessed the expression of SOX9, a marker of airway SMG progenitors that have been previously shown to participate in SAE regeneration (15, 27, 28). As expected, all three of these targets were upregulated in both severely injured and moderately injured epithelia, with the level of upregulation corresponding to the level of injury (Figure 6D). We additionally verified that the proportion of basal cells (KRT5+) and non-basal cells (KRT8+) was similar between the treatment groups at the time of RNA collection by immunofluorescent staining (Figures 6E–G). Consistent with the published literature (29) and our prior observations (15, 16), we noted that epithelial cells on the airway surface were larger and incorporated EdU more frequently in explants that were injured versus uninjured (Figures 6E–G).

### Design of the 3D-printed explant holding chamber for long-term culture and live imaging

**Figure 7:**
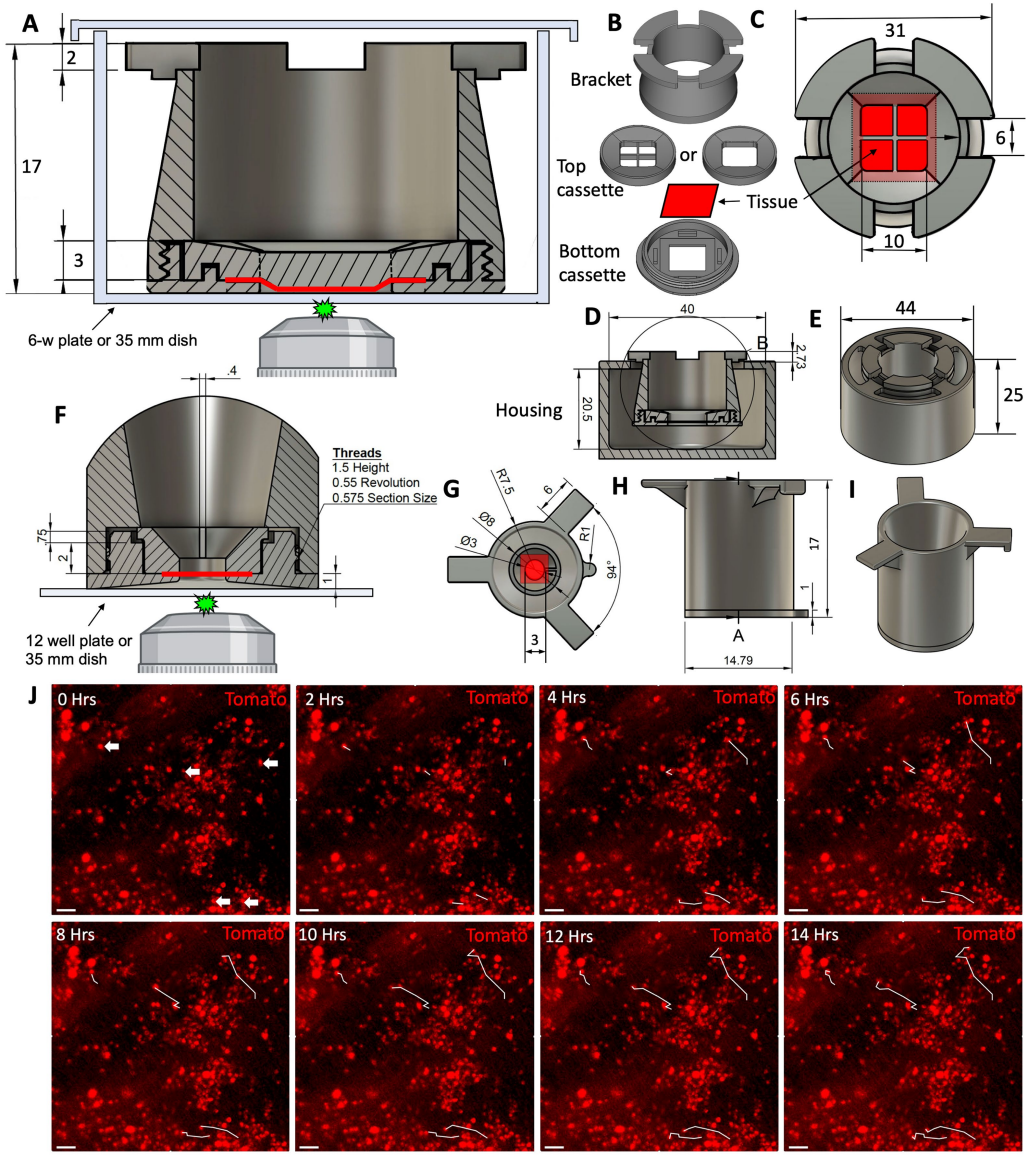
Design of the 3D-printed tissue culture chamber for live cell imaging. **(A)** Cross section of the tissue-transwell placed into a tissue culture plate for live imaging. The epithelial tissue sample is depicted in red. **(B)** Individual components of a disassembled tissue-transwell. **(C)** Top view of a loaded and assembled tissue-transwell. **(D)** Cross section of the tissue-transwell inside its external housing chamber. **(E)** 3D overview of the tissue-transwell inside the external housing chamber (loose-fitting lid is not shown). **(F–I)** A smaller version of the tissue-transwell designed for imaging samples from smaller animals (i.e., mice and rats). **(F)** Cross section of the insert with tissue positioned for live imaging. **(G)** Top view of the tissue-transwell insert. **(H)** Side view of the insert. **(I)** 3D view of the insert. **(J)** 14 h time lapse of Tomato+ ferret SAE on day 7 after engraftment on WT non-fluorescent trachea, white tracks showing the path that the cell had completed at each timepoint. Scale bars are 50 μm.

One of the greatest limitations of mammalian *in vivo* systems is the difficulty of performing high-resolution live imaging (30). An *ex vivo* culture system has brought us a step closer to circumventing this limitation. Existing methods for live imaging of the tracheal explants involve pinning the tissue to the support stage and imaging it under a dissecting microscope. This method works well for terminal assays such as mucociliary clearance, but is unsuitable for long-term culture of the explants. Additionally, the resolution and magnification of a dissecting microscope is limited even though individual cells can be resolved. For these reasons we sought to develop an explant culture chamber in which tissue can be positioned for imaging with a confocal laser scanning microscope while sterility is maintained. We designed and 3D-printed a 3-piece device that can keep epithelial tissue explants such as tracheas at an ALI and position them for confocal imaging (Figures 7A–I). Conceptually, an assembled device loaded with an explant resembles a transwell™ (Corning). Therefore, we will refer to it as a “tissue-transwell” for simplicity. The key difference between our device and traditional transwells that are available for *in vitro* culture is that instead of the permeable support for cells at the bottom, the tracheal explant itself fills the opening at the bottom of tissue-transwell acting as a barrier between the two compartments (which can be filled with media or be exposed to air). During assembly of the tissue transwell, the tissue is placed between the top and bottom parts of the cassette and this “sandwich” is screwed into the bracket (Figure 7B). The loaded tissue-transwell can be cultured in a 6-well dish or in a special 3D-printed housing chamber. Different sizes of this device were designed for mouse/rat, ferret, and human explants and printed using biocompatible plastic Med 610 HQ (31). After autoclaving the devices, we loaded them with fully brushed WT ferret tracheas and seeded the lumen with 2*10^6^ Tomato+ ferret airway basal cells. Seven days later we performed live imaging of the explants, confirming that this system is compatible with live imaging and that the newly engrafted cells were viable and able to migrate on day 7 after seeding (Figure 7J).

### Basal cells engrafted *ex vivo* in tissue-transwells can differentiate into ciliated and secretory cells within 21 days of ALI culture

**Figure 8:**
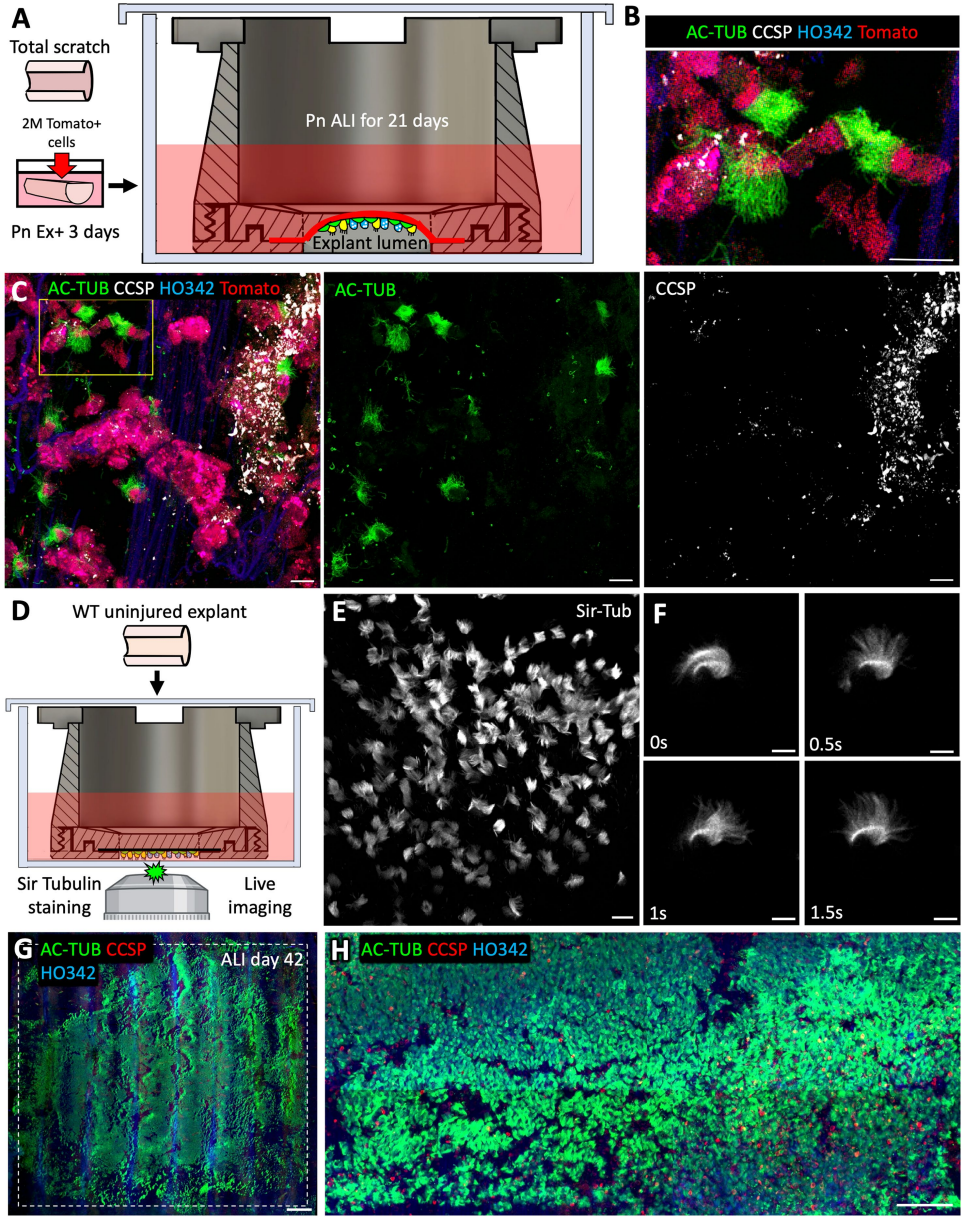
After 21 days of ALI culture, *ex vivo* engrafted basal cells can differentiate into ciliated and secretory cells. **(A)** Experimental design: Freshly collected tracheal explants from adult WT ferrets were fully brushed to remove all the native SAE and then seeded with 2*10^6^ p6 Tomato+ ferret SAE cells. After engraftment, explants were cultured submerged in Pneumacult Ex+ medium for 3 days prior to being loaded into tissue-transwells and cultured in Pneumacult ALI medium with an air bubble trapped in the explant lumen to ensure ALI. Samples were collected after 21 days of differentiation at ALI. **(B,C)** Confocal micrographs of Tomato+ engrafted cells on ALI day 21 showing the presence of AC-TUB+ ciliated cells and CCSP+ secretory cells. **(D)** Experimental design: WT uninjured tracheal explant was placed into a tissue-transwell (with a cross-piece) and incubated in Pneumacult ALI medium containing Sir Tubulin reagent to stain the motile cilia. **(E,F)** Confocal micrographs of beating cilia. **(G,H)** Confocal micrographs of WT uninjured explants that were loaded into tissue-transwells (without the cross pieces) and cultured at an ALI for 42 days. Scale bars are: 20 μm in **(B,C,E)**; 10 μm in **(F)**; 1,000 μm in **(G)**; and 100 μm in **(H)**.

The main purpose of designing the tissue-transwell was to open the tracheal tube and position the airway lumen near the microscope objective for imaging. A secondary purpose was to compartmentalize the luminal and adventitial sides of the explant so that they can be treated with different media. We made two customizable versions of the top tissue cassette piece: one with a 1 cm^2^ opening and another one with a thin cross-piece subdividing this opening into 4 smaller ones. In the version with the larger opening, the curvature caused by the tracheal cartilage is retained and an air bubble is trapped underneath, creating an ALI. In the version with a cross-piece, the tissue is held flat against the bottom of the dish for imaging. Tissue culture in the appropriate media at an ALI can stimulate differentiation of airway epithelia after basal cell engraftment or after an injury that removes differentiated luminal cells. As a proof of concept, we completely removed the native SAE by brushing and seeded it with Tomato+ SAE cells prior to transitioning the explant to an ALI. We used a version of the tissue-transwell with a 1 cm^2^ opening without the cross piece in the middle (Figure 8A). Having a trapped air bubble ensures that the ALI forms even when the explant is loaded imperfectly or when the tissue is accidentally punctured. After 21 days of differentiation at an ALI, the engrafted Tomato+ ferret SAE cells differentiated into AC-TUB+ multi-ciliated cells and CCSP+ secretory cells, even without reaching 100% confluency (Figures 8B–D). On the other hand, loading the explants into the version of the tissue-transwell with a cross piece and flattening them for imaging made it possible to image cilia motility live after adding Sir Tubulin (a reagent for viable labeling of the cilia; Figures 8E,F). Finally, we evaluated how well the tracheal explants tolerate long-term culture at an ALI within the tissue-transwell. In these experiments, the explants were exposed to Pneumacult ALI medium on the adventitial side for 42 days (with media exchanges every 2–3 days). In this experiment, most of the SAE cells in the transwell opening were ciliated and secretory (Figures 8G,H), suggesting that the device can be used for *ex vivo* modeling of acute and chronic lung and airway diseases.

## Discussion

The current study provides proof-of-principle evidence that *ex vivo* tracheal injury and culture can be effectively utilized to address biological questions regarding the dynamics of epithelial regeneration in the airway. The whole-mount staining and tissue clearing protocol that we describe here allowed us to rapidly evaluate large areas of the SAE and SMGs and can be used to efficiently capture low-frequency events. For example, we were able to assess the abundance of ionocytes on multiple whole ferret tracheas (manuscript is under review) and to survey transgenic cell engraftment. Additionally, this method of staining revealed a previously underappreciated complexity of airway SMG structure, vascularization, and innervation (Figure 4). This protocol can be adapted to other organs, as our colleagues have used it successfully for staining the pancreas, spleen, and adipose tissue (unpublished data).

The current study also provides information on suitable culture conditions for *ex vivo* injury experiments. Our discovery that acceptable regeneration was achieved in F-medium containing Y-27632 (Figures 1, 5) and that this was significantly more effective than simple DMEM (Figure 5), leads us to expect that one or more of the F-medium additives supports tracheal regeneration *ex vivo*. Our demonstration that Y-27632 stimulates robust repair of the explant airway surface after injury *ex vivo* (Figure 1) is interesting given that one of the well-described functions of Y-27632 is to prevent the induction of stem cell death by dissociation (anoikis) (32). This leads us to speculate that Y-27632 protects the pioneer airway basal stem cells that detach from the main cell mass in their attempt to repair the injured regions. The effects of other components of F-medium on regeneration can be further tested by removing them individually in future studies. For the purposes of this study, we proceeded with F-medium given that it resulted in satisfactory regeneration of the tracheal epithelium by progenitors from both the SMGs and SAE. Our recent work indicates that under these media conditions fully brushed explants gradually re-epithelialize ~90% of the explant surface within 15 days of the injury and majority of this regeneration is attributed to SMG progenitors (16).

Our study of EdU incorporation rates demonstrates that SMGs are non-uniform in their injury responses, with about 25% of the gland clusters abundantly incorporating EdU following injury, often concomitant with strong KRT5 expression in gland ducts (Figure 1). Our observation that EdU was more frequently incorporated into the gland ducts than in the acini is consistent with previous studies (15), and this could be due to their closer proximity to the injured airway surface. On day 5 after the total SAE removal, the airway surface overlying ‘active’ gland clusters was locally covered with EdU+ basal cells that were contiguous with the gland ducts suggesting that these glands are the first ones to start populating the airway surface after injury (Figure 1). It is interesting that when fully brushed explants were cultured in the continuous presence of EdU for 9 days (rather than being pulsed with it for 24 h on day 3), only about 37% of the gland acini contained a high abundance of EdU+ cells, the other 63% having a relatively sparse EdU+ cell density (Figure 1). In future, it will be important to determine whether the observed mosaic pattern of injury-induced SMG proliferation occurs *in vivo* and whether specific gland clusters are primed to respond to injury more intensively than others. Alternatively, the observed mosaic pattern of actively regenerating SMG clusters might reflect a mechanism whereby a gland that is first to engage in regeneration inhibits proliferation of progenitors in the neighboring glands. Functionally, this could prevent depletion of all the reserve gland progenitors at once. Indeed, gland progenitors are depleted in bronchiolitis obliterans syndrome (BOS) following lung transplantation (17, 18), which could reflect that this mechanism is broken in this form of chronic lung allograft dysfunction.

Investigation of airway injury resolution mechanisms is ultimately aimed at developing therapies for various lung diseases. Many cell-based therapies rely on delivering *in vitro*-expanded stem/progenitor cells that have been genetically modified. For example, cell therapies that lead to engraftment of gene-corrected autologous airway stem cells in the patient could benefit about 10% of *CF* patients with nonsense variants of CFTR. Similar cell therapeutic strategies can be applied to patients with other chronic lung diseases. For example, depleted airway epithelial stem cell niches can be reconstituted using patient-derived iPCSs which would mediate continuous large-scale tissue repair later on (33). Efficient cell engraftment remains challenging *in vivo* (13), and the tractable system we provide for optimizing engraftment conditions can help to overcome this barrier. As a testament to this expectation, we demonstrate that only limited engraftment occurred in intact airway epithelia, but the efficiency was greatly improved when the cells were seeded onto a partially of fully denuded tracheal grafts rather than onto intact explants (Figures 2, 3). Similar observations were made in a mouse nasal cell transplant model previously (34). The reason for it might be a better access of the engrafted cells to the ECM of the basement membrane where they can attach. This is encouraging, considering that the airways of patients who would most benefit from cell therapies are often in a chronically injured state. Another possibility is that intact airway epithelium has too much locomotion for exogenous cell to engraft due to ciliary beating. In the future, low-throughput *ex vivo* screens can be used after high-throughput *in vitro* screens to validate the potential of candidate compounds to enhance engraftment of airway stem cells. Good potential targets for such screens could be the drugs that temporarily stall ciliary beating, mucolytics, and tight junction modulators.

In optimizing cell engraftment, it is important to understand what changes cells undergo when they are moved from tissue culture plastic to a complex scaffold such as a partially denuded trachea. Specifically, the observed changes in cell adhesion markers provide insights into which pathways to target to improve the efficiency of cell engraftment. In this work we compared the expression of several cell adhesion markers between the cells engrafted onto tracheal scaffolds and the cells seeded on ECM-coated plastic. Overall, we observed a decline in the expression of various integrins (ITGB1, ITGA6 and ITGA3), but not in CD44 (Figures 6A,B). We speculate that that this downregulation of integrin expression led to a diminished attachment of engrafted cells to the explants compared to the tissue culture plates. If this speculation is experimentally validated, it is possible that the airway stem cell engraftment *ex vivo* and *in vivo* might be improved by stimulating integrin expression.

Enhancement of our general understanding of airway epithelial regeneration holds promise for the advancement of cell therapies. Multiple studies have evaluated global transcriptional changes that are associated with wound healing in the epidermis and airways *in vivo* (25, 35, 36). In this work, we validated some of these observations in our *ex vivo* system on day 14 after a tracheal brush injury. We detected an upregulation of genes involved in ECM deposition and remodeling in regenerating SAE, specifically transcription of MMP9 and LAMA3 was significantly increased in injured conditions (Figure 6D). This had been previously observed at the leading edges of regenerating mouse epidermis after a punch biopsy *in vivo* (25). Additionally, MMP9 is elevated in the airway epithelial cells of human patients who develop BOS after lung transplantation (26, 37, 38). Also, our group and others had previously shown that SOX9+ glandular progenitors participate in SAE repair after severe injury *in vivo* and *ex vivo* (14, 15, 27). Our observation that SOX9 mRNA expression is elevated in regenerated SAE on DPI14 is consistent with those findings (Figure 6D). Thus, our *ex vivo* tracheal injury model can reliably capture transcriptional changes in the airway epithelium during wound healing. In the future, the field would benefit from unbiased surveys of global changes at the transcriptional and protein levels within the airway epithelium following injury *ex vivo* and *in vivo*.

Lastly, we designed and 3D-printed a reusable tissue-transwell (Figure 7) that can be used for a variety of applications. For example, it can be used to culture airway explants at an ALI, where the airway lumen is exposed to air while the adventitia is in contact with medium (Figure 8). These culture conditions can be used to model airway diseases, because they reflect the physiological airway environment more accurately than submerged explant cultures do. Moreover, the ability to mount whole tissues for live imaging is expected to be helpful in numerous other applications, notably for recording the motility of cilia (Figure 7), clearance of the mucus, tracing cell lineages and evaluating migration during wound healing (Figure 6). Although this *ex vivo* model has advantages over *in vitro* culture, it is important to recognize its limitations relative to *in vivo* systems, for example the lack of innervation and vascular perfusion, as well as hypoxia, a condition that is unavoidable in the absence of constant air flow for gas exchange. Researchers need to be aware that *ex vivo* conditions might alter injury responses, and thus there may be discrepancies between *ex vivo* and *in vivo* systems, which emphasizes the importance of cross-system validation of biological findings.

## Conclusion

In this work we demonstrate that *ex vivo* ferret tracheal cultures can be useful to answer certain biological questions that are challenging to address *in vivo* or *in vitro*. We also characterize the tools and approaches needed in order to take full advantage of this tracheal explant model, namely whole-mount tissue staining, *ex vivo* mechanical injury, stem-cell engraftment, and tissue-transwells for live imaging and ALI culture. In the future, this explant model can be used to explore changes associated with wound healing in airway epithelia and optimize cell engraftment conditions. The tissue-transwells that we introduce here are cost-effective and reusable, and they can be applied to a variety of membrane-containing organs including airways, esophagus, stomach, intestine, bladder, and skin. We encourage investigators to contact us for details on where to acquire tissue-transwells for their research applications.

## Data availability statement

The raw data supporting the conclusions of this article will be made available by the authors, without undue reservation.

## Ethics statement

The animal study was reviewed and approved by The University of Iowa Institutional Animal Care and Use Committee (IACUC).

## Author contributions

VI, AP, JE, and KP designed the research studies. VI and AP conducted experiments and acquired data. VI, AP DD, SK, and KP designed the tissue-transwell. VI, JE, and KP analyzed data. VI wrote the manuscript. VI, JE, KP, KF, TL, and CG edited the manuscript. All authors contributed to the article and approved the submitted version.

## Funding

This work was funded by the following grants: R01 HL136370 (to KP); P01 HL152960 (to JE), NHLBI Contract 75N92019R0014 (to JE); P30 DK054759 (to JE), R01 HL165404 (to JE); T32 HL007638 (to TL), K99HL155843 (to TL).

## Conflict of interest

The authors declare that the research was conducted in the absence of any commercial or financial relationships that could be construed as a potential conflict of interest.

## Publisher’s note

All claims expressed in this article are solely those of the authors and do not necessarily represent those of their affiliated organizations, or those of the publisher, the editors and the reviewers. Any product that may be evaluated in this article, or claim that may be made by its manufacturer, is not guaranteed or endorsed by the publisher.
